# Investigating the dark-side of the genome: a barrier to human disease variant discovery?

**DOI:** 10.1186/s40659-023-00455-0

**Published:** 2023-07-20

**Authors:** Niamh M. Ryan, Aiden Corvin

**Affiliations:** Neuropsychiatric Genetics Research Group, Department of Psychiatry, Trinity College Dublin, Dublin, Ireland

**Keywords:** Short-read sequencing, Dark regions, Gene-disease associations

## Abstract

The human genome contains regions that cannot be adequately assembled or aligned using next generation short-read sequencing technologies. More than 2500 genes are known contain such ‘dark’ regions. In this study, we investigate the negative consequences of dark regions on gene discovery across a range of disease and study types, showing that dark regions are likely preventing researchers from identifying genetic variants relevant to human disease.

**Supplementary Information:**

The online version contains supplementary material available at 10.1186/s40659-023-00455-0.

## Background

Genome-wide association studies (GWAS) have successfully identified regions of the genome associated with human diseases, but have been less successful at determining the contributory variants involved. Similarly, many genes have been identified as biologically relevant candidates for disease (through linkage studies, gene and protein expression studies, animal models, etc.), but without corresponding support for risk variants from genomic sequencing. Whether these genes are false positives, or this missing heritability is driven by something else remains unexplained.

As understanding of the structure and sequence of the human genome improved it has become apparent that there are regions of the genome that are difficult, or impossible, to assembled or aligned using next generation short-read sequencing (SRS) methods [1–3]. Ebbert et al. described these regions as “dark regions” of the genome (either “dark-by-depth”, with few mappable reads or “dark-by-alignment”, caused by duplicated sequences and multi-mapping reads) [4]. Estimates of how much of the genome is dark vary by definition of what constitutes a dark region; study design; and the sequencing technology employed. For standard whole genome SRS, the proportion of the genome that is dark or difficult-to-map is estimated to range between 84 and 145 Mb [1, 4], with 748 to 2512 protein-coding genes reported as being at least partially dark [2, 4]. The number of dark genes implicated in human disease similarly varies by study and the database of disease-genes used. Ebbert reported 76 disease-associated genes from the public HGMD database (2012 version) as overlapping dark regions [4], while Mandelker identified 464 medically relevant dark genes, based on ACMG guidelines and ClinVar data (2012 version) [2]. While these numbers may be under-estimates due to the age of the databases used, it is clear that a non-trivial proportion of the genome is dark and that this may be an obstacle to the discovery of risk mutations relevant to human disease.

## Results and discussion

To date, no-one has looked at the potential impact of dark regions on gene discovery, likely in part due to the difficulties of investigating null-findings or the absence of data. The aim of this analysis was to investigate whether dark regions could affect our ability to identify disease-relevant variants, both when fine-mapping genome-wide significant GWAS loci and when performing whole exome (WES) or whole genome (WGS) sequencing studies.

We investigated the overlap between a curated list of dark regions and dark genes from Ebbert et al. [4], against annotated GWAS loci, here-on referred to as Genomic Risk Loci (GRL), for eight different diseases and complex traits: autism spectrum disorders (ASD); schizophrenia (SCZ); body mass index (BMI); bipolar disorder (BD); major depressive disorder (MDD); cholesterol; amyotrophic lateral sclerosis (ALS); and Crohn’s disease. These eight GWAS were taken from the FUMA public database of GWAS studies and each GRL was annotated with summary information for all genes in LD (R > 0.6) with the tagging SNP [5]. Across the eight studies, 33–73% of GRLs contained dark regions (Table 1). The amount of dark sequence within these regions varied from 92 bp (ASD) to more than 1 Mb (SCZ and BMI). Furthermore, 7–20% of the genes at each locus were found to overlap dark regions, with up to 2.5% of these genes having dark-CDS regions (dark protein-coding regions).

**Table 1:** Summary of dark regions overlapping genome-wide significant loci from GWAS studies

GWAS Disorder	FUMA ID	Genomic risk loci (GRL)				Mapped genes (MGs)				# MGs with dark CDS			GO enrichment			
		# GRLs	# GRLs overlapping DRs	% GRLs overlapped by DRs	Overlap (bp)	# MGs	# MGs overlapping DRs	% MGs overlapping DRs	Overlap (bp)	Any	> 5%	> 20%	All dark GWAS MGs vs Genome	Relevant Gene Ontology enrichment terms (biological processes)	GWAS MGs with Dark CDS vs remaining GWAS	Relevant Gene Ontology enrichment terms
ASD	10	3	1 GRL (1 DR)	33	92 bp	113	8 (37 DRs)	7	12 Kb	0	0	0	106 terms (p < 0.05)	Neuron differentiation, ERBB signalling pathway	–	–
SCZ	323	242	109 GRL (1617 DRs)	45	1075 Kb	879	176 (962 DRs)	20	433 Kb	21	15	10	17 terms (FDR sig)	Terms related to neurogenesis; synapse (structure and signalling); and calcium ion transport	6	Striatal medium spiny neuron differentiation; striatum development; sub-pallium development; forebrain neuron differentiation; and forebrain generation of neurons
BMI	221	832	430 GRL (3427 DRs)	52	1317 Kb	8663	1545 (5322 DRs)	18	1902 Kb	133	83	41	228 terms (FDR sig)	Terms related to cell morphogenesis, development and differentiation; signalling, signal transduction and cell communication; cell migration and motility, localization, cell adhesion and cell junction organization; neurogenesis, neuron development and differentiation; and immune response	35	SMAD protein phosphorylation; sex determination; steroid biosynthesis; muscle morphogenesis; transmembrane receptor protein serine/threonine kinase signaling pathway; and GABAergic synaptic transmission
BD	192	16	9 GRL (157 DRs)	56	77 Kb	293	45 (168 DRs)	15	86 Kb	6	5	3	160 terms (p < 0.05)	Terms related to synaptic structure and function plasticity; action potential; central nervous system and brain development; and myelination	–	–
MDD	191	51	22 GRL (144 DRs)	43	99 Kb	645	116 (426 DRs)	18	121 Kb	8	3	0	45 terms (FDR sig)	Terms related to neurogenesis; synaptic function; neurotransmitter transport; and nervous system development	40	Presynaptic active zone assembly; protein localization to synapse; pre-synapse organization; and pre-synapse assembly
Cholesterol	113	56	28 GRL (130 DRs)	50	49 Kb	285	42 (106 DRs)	15	35 Kb	6	3	2	433 terms (p < 0.05)	Phospholipid homeostasis; reverse cholesterol transport; monocarboxylic acid biosynthetic process; and carboxylic acid metabolic process	–	–
ALS	423	11	8 GRL (41 DRs)	73	16 Kb	141	18 (46 DRs)	13	21 Kb	2	2	1	185 terms (p < 0.05)	Membrane trafficking, signal transduction and ion channel transport; autophagy; central nervous system and oligodendrite development; dopaminergic neuron differentiation, neuron maturation, regulation of cell differentiation; central nervous system myelination and axon ensheathment and neuron axonogenesis	–	–
Crohn's disease	2	71	27 GRL (470 DRs)	38	212 Kb	276	47 (228 DRs)	17	97 Kb	7	5	5	438 terms (p < 0.05)	Terms related to immune response; cytokine activity; receptors and signalling; and glutamine metabolism	–	–

Summary of annotated GWAS datasets from FUMA and their overlap with dark regions. Showing: GWAS Disorders and complex traits (ASD: autism spectrum disorders; SCZ: schizophrenia; BMI: body mass index; BD: bipolar disorder; MDD: major depressive disorder; ALS: Amyotrophic lateral sclerosis); FUMA ID; total number of genomic risk loci (GRL); number of GRL overlapping dark regions (and the number of dark regions overlapping GRLs); % of GRLs that are dark; the combined length of GRLs that are overlapped by dark regions; the total number of mapped genes in GRLs; the number of MGs overlapping dark regions (and the number of dark regions overlapping MGs); the combined length of the MGs that are dark; the number of MGs that have dark coding regions (CDS): any, more than 5% dark-CDS, more than 20% dark-CDS; results of gene ontology enrichment (GO) analysis (biological processes) for all GWAS dark genes vs the rest of the genome, with the number of enriched terms (FDR corrected or p-value < 0.05); summary of enriched GO terms related to the phenotype; gene ontology enrichment (GO) analysis (biological processes) for GWAS MGs with dark-CDS vs the remaining GWAS MGs (for the GWAS that FDR significant terms from all dark MGs vs genome)

While only a small percentage of GWAS genes are affected by dark-CDS, it is not expected that all genes at each GRL will play a role in disease aetiology, as demonstrated by fine-mapping, pathway analysis and other downstream analyses of GWAS data [6, 7]. To assess their potential functional impact, the genes with dark regions were investigated for enrichment for biologically relevant gene ontology (GO) terms [8]. All eight sets of dark GWAS genes were enriched for GO terms previously associated with their corresponding disease and trait (Table 1, Additional file 1). In particular, the dark genes from the SCZ, BMI and MDD GWAS studies (the GWAS with the greatest number of GRL genes) returned FDR-significant GO terms. For these three datasets, a comparison of the dark GRL genes against the remaining (not-dark) GRL genes further refined the biological relevance of the GO terms identified (p-value < 0.05, but not FDR-significant) (Additional file 1). In summary, GWAS dark genes and dark-CDS genes are enriched for biologically relevant GO terms, suggesting there are biologically relevant genes in regions of the genome significantly associated with disease that are not fully accessible to SRS technology. Therefore, fine-mapping studies may fail because the pathogenic variants are in dark regions and cannot be accessed.

To investigate the impact of dark regions on the discovery of rare variant associations from WES studies we looked at the overlap of dark regions with the protein-coding regions of genes from the Schizophrenia Exome Sequencing Meta-analysis (SCHEMA) consortium and the Autism Exome Sequencing consortium (ASC). Despite the size of the SCHEMA cohort (24,248 cases and 97,322 controls), only ten genes were found by the authors to be significantly associated with SCZ [9]. Of these ten, only TRIO has a partially dark-CDS (CDS 0.4% dark). Extending the search space to include all genes from SCHEMA with p-value < 0.05 (928 genes), 222 had partially dark gene bodies (including non-coding regions and introns); 22 have partially dark-CDS, ten with > 5% dark-CDS. Of these ten, six have supporting evidence from the literature of having a neuro-developmental or psychiatric function (Additional file 1).

Of the 102 putative ASD-associated genes identified by the ASC (FDR < 0.1) [10], four have dark-CDS, with *CORO1A* and *SHANK3* being more than 5% dark (Additional file 1). Of these 102 genes, 101 are annotated by SFARI Gene 3.0 [11] as Score 1 (High Confidence ASD gene), with one gene being Score 2 (Strong Candidate). Across the full set of SFARI genes we found an enrichment of dark regions in Score 2 and Syndromic (ASD with co-morbid phenotypes) genes with ASC q-values > 0.3, suggesting that some candidate genes for ASD may not perform well in genetic association studies due to their gene bodies being partially dark to sequencing (Additional file 1: Fig. S1).

**Fig. 1:**
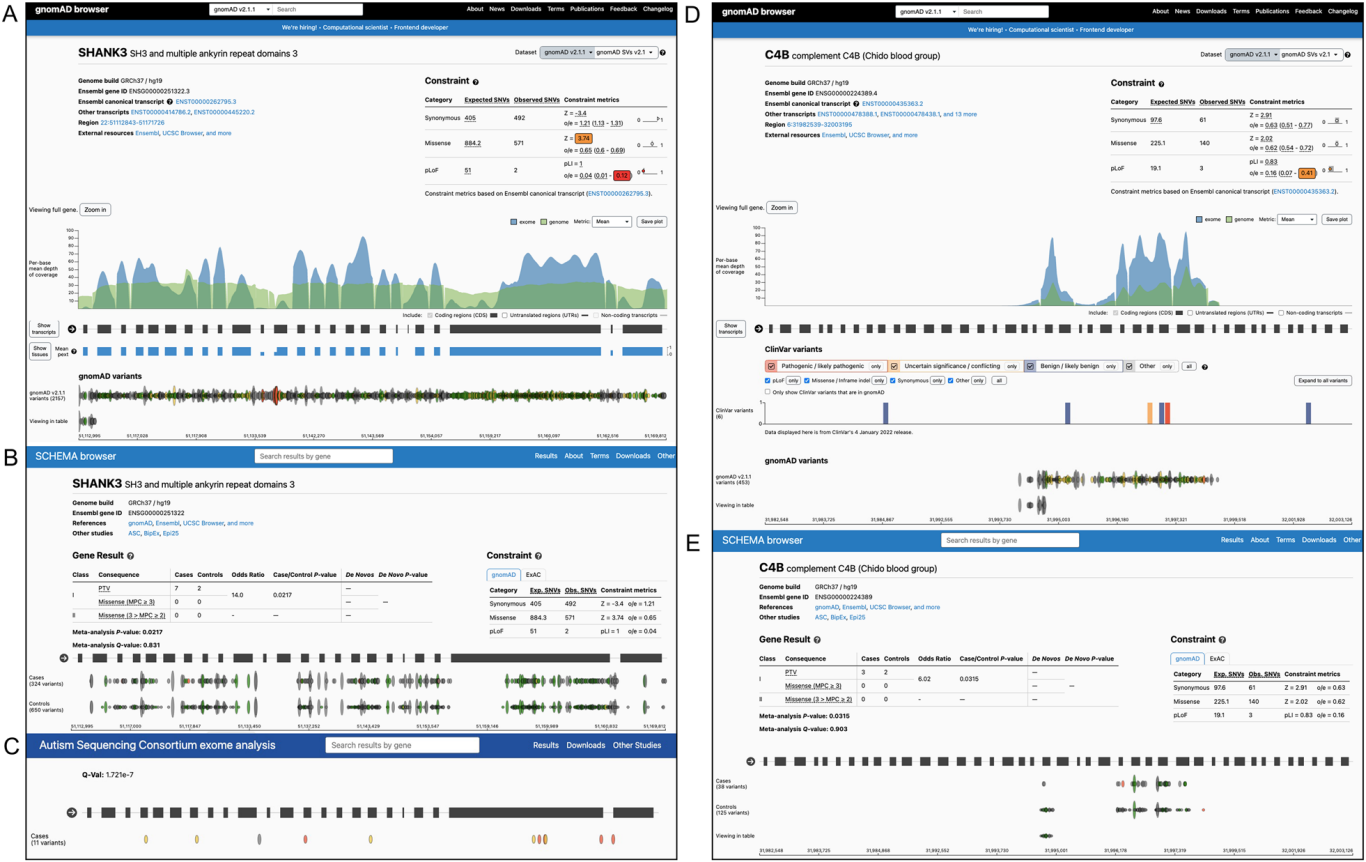
Examples of two genes affected by dark regions overlapping their CDS, showing modified browser views of SHANK3 and C4B from GnomAD Browser of human genetic variation (showing the average read depth of both whole exome and whole genome sequencing data); SCHEMA Browser of SCZ associated rare variants (and for SHANK3, the Autism Sequencing Consortium Browser of rare variants). Note for each browser the conspicuous absence of any genetic variants (pathogenic or benign) from low read-depth (dark regions) from exome and whole genome sequencing data

Two examples of dark candidate disease genes from SCHEMA and ASC are SHANK3 and C4B, shown in Fig. 1. SHANK3 is a top hit from ASC, nominally-associated in SCHEMA, and has also been implicated by common variant GWAS for schizophrenia [12]. As can be seen in Fig. 1, the coding regions of SHANK3 are 7.7% dark and WES in particular is unable to identify genetic variants from 5 different exons. Many studies have supported SHANK3’s role in both SCZ and ASD [13–16]. C4B was also found to be within the nominally-significant SCHEMA gene set and is a SFARI Score 2 gene. Figure 1 shows that C4B is substantially dark (73% dark-CDS), preventing the discovery of genetic variants across most exons. Both C4B and its paralog C4A (also ~ 74% dark-CDS) have been suggested to play a role in SCZ [6, 17–19]. These examples support the theory that candidate disease genes overlapping dark regions may contain rare variants that are not accessible to SRS technology and thus are missed when calculating gene-disease associations.

Ebbert et al. [4] showed that dark genes are involved in many diseases including neuropsychiatric disorders. We have confirmed this and given evidence of even more neuro-psychiatric genes affected by dark regions. As this analysis is based on a conservative number of dark regions and dark genes (749 genes) we propose that we have reported the lower- rather than upper-limit of potential disease-associated genes affected by dark regions. However, it should also be noted that the number of dark regions, both within genes and intragenic regions, vary dramatically depending on both the technology and genome build used. Longer read lengths (Illumina 250 bp) have up to 35% less dark regions than shorter read lengths (Illumina 100 bp), as longer reads map more uniquely than shorter reads [4]. GRCh38 appears to have up to three-fold greater proportion of dark regions than GRCh37 for all read lengths, possibly due to the inclusion of alternative contigs and additional halpotypes from heterozygous regions, which increases the amount of non-unique sequences from SRS in the GRCh38 + alternative contigs reference assembly than the GRCh37assembly [4]. Thus both read length and genome reference build appear to be important factors for the proportion of dark regions present in SRS WGS data.

This study makes use of publicly available GWAS data from FUMA. Larger, better-powered GWAS have since been performed for a number of these diseases, identifying an even greater number of GRLs, each likely to also contain dark regions overlapping putative risk genes. Despite these limitations, we have shown that dark regions overlap with genome-wide significant GWAS loci across a range of traits and disorders, affecting as much as 1.3 Mb of sequence under these peaks and that the genes with dark regions are enriched for biologically relevant GO terms, showing they are relevant to disease-risk. Care must be taken when fine-mapping GWAS regions as the causal variants may be located in regions that are dark to SRS and will therefore be missed. A similar issue can be seen when looking at rare variant association studies. From our analysis, dark regions are likely to contribute to missing heritability.

There needs to be greater awareness of the potential effects of dark regions when using SRS to investigate both common and rare genetic variants contributing to disease. Genes of interest maybe partially inaccessible to the technology being used, meaning that variants at these locations cannot be identified using standard protocols. To overcome this, short-read WES and WGS data can be re-analysed using alignment methods specifically developed to correctly align ambiguous reads (such as from camouflaged regions, repetitive sequences, insertions and deletions) and successfully map non-unique sequences which would normally be discarded [4, 20, 21]. Furthermore, long read sequencing technologies (such as PacBio and ONT) have been shown to reduce the amount of dark gene-body regions by up to 77% [1, 3, 4]. The most recent reference assembly, T2T-CHM13, was generated using a combination of PacBio HiFi and Oxford Nanopore ultralong-read sequencing and represents the first complete genome [22], including the 8% of the genome that has remained hidden since the first human reference genome was published in 2000 [23]. LRS could therefore be used to re-investigate dark genes with evidence of disease effects from other studies (such as animal knock-out models, protein expression studies, etc.). However, limitations of LRS technologies need to be addressed before this technology can be generally adopted [24]. LRS is currently more expensive than SRS, though the costs are fast coming down. Library preparation is less forgiving than for SRS as fresh material or even intact cells are recommended to minimise degradation of ultra-long high molecular weight DNA (which also requires specialised DNA isolation protocols). Both PacBio and ONT have higher error rates for SNV detection compared to SRS, though LRS have been shown to be better at calling SNVs in problematic areas [3]. There is less choice of tools for both raw data analysis as well as mapping and variant calling tools for LRS than SR-NGS but are constantly being improved [25].

## Conclusion

Only roughly 85–92% of the genome can be sequenced confidently using SRS technologies [1], meaning ~ 10% of the genome is inaccessible or “dark” to SRS. We have investigated the negative consequences of dark regions on gene discovery across a range of disease and study types, showing that dark regions are likely preventing researchers from identifying genetic variants relevant to human disease. This suggests dark regions are likely to contribute to missing heritability. Long read sequencing can be used to investigate these dark regions and aid the discovery of pathogenic variants that we currently cannot identify using SRS technology.

## Methods

The detailed methods can be found in the Additional file 1.

## Supplementary Information

**Additional file 1: Methods S1.** **Table S1.** GO enrichment analysis for the Schizophrenia dark genes vs genome generated 17 FDR significant GO terms. **Table S2.** GO enrichment analysis for the BMI dark genes vs rest of the genome generated 228 FDR significant GO terms, of which the top 30 are presented in this table. **Table S3.** GO enrichment analysis for the MDD GWAS genes with dark regions vs rest of the genome returned 45 FDR significant GO terms, of which the top 30 are represented in this table. **Table S4.** Results of the GO enrichment for the Cholesterol GWAS dark vs rest of genome, showing the top 30 enriched GO terms (out of 433 terms with p-value< 0.05, but not FDR significant). **Table S5.** Results of the GO enrichment for the Crohn’s dark vs genome, showing the top 30 enriched GO terms (out of 438 terms with p-value< 0.05, but not FDR significant). **Table S6.** Results of the GO enrichment for the ASD dark vs genome, showing the top 30 enriched GO terms (out of 106 terms with p-value< 0.05, but not FDR significant). **Table S7.** Results of the GO enrichment for the ALS GWAS dark genes vs rest of the genome, showing the top 30 enriched GO terms (out of 185 terms with p-value< 0.05 but not FDR significant). **Table S8.** Results of the GO enrichment for the BD GWAS dark genes vs rest of the genome, showing the top 30 enriched GO terms (out of 160 terms with p-value< 0.05 but not FDR significant). **Table S9.** SCZ darkCDS vs genome. **Table S10.** Results of the GO enrichment for the Schizophrenia dark CDS vs remaining GRL genes. No terms were FDR significant, however, five terms with p< 0.05 were returned, all related to brain development. **Table S11.** BMIdarkCDS vs genome **Table S12.** Top 20 GO terms for the BMI dark CDS vs remaining GRL genes. No FDR significant terms, however, 35 terms with p< 0.05 were returned. **Table S13.** MDDdarkCDS vs genome. **Table S14.** Top 20 GO terms for the MDD GWAS genes with dark CDS vs remaining GWAS genes. In total 40 terms (p< 0.05 but not FDR significant) were returned. **Table S15.** All 22 SCHEMA genes with p<0.05 with at least partially dark CDS regions of which ten genes have >5% dark CDS. **Table S16.** Subset of ASD associated genes from the ASC which have dark CDS regions, of which two genes have >5% dark CDS. **Table S17.** SFARI Score 1 (High Confidence) genes with dark CDS regions, of which four genes have >5% dark CDS. **Figure S1.** Modified browser views of *RAC3* from A. GnomAD Browser of human genetic variation (showing the average read depth of both whole exome and whole genome sequencing data) and B. SCHEMA Browser of SCZ associated rare variants. Note for each browser the conspicuous absence of any genetic variants (pathogenic or benign) from low read-depth (dark) regions from exome and whole genome sequencing data, in particular for exon 1. **Figure S2.** Modified browser views of *TRAPPC10* from A. GnomAD Browser of human genetic variation (showing the average read depth of both whole exome and whole genome sequencing data) and B. SCHEMA Browser of SCZ associated rare variants. **Figure S3.** Modified browser views of *UBE2L3* from A. GnomAD Browser of human genetic variation (showing the average read depth of both whole exome and whole genome sequencing data) and B. SCHEMA Browser of SCZ associated rare variants. Note for each browser the conspicuous absence of any genetic variants (pathogenic or benign) from low read-depth (dark) regions from exome and whole genome sequencing data, in particular for exon 1. **Figure S4.** Modified browser views of *FAM86B1* from A. GnomAD Browser of human genetic variation (showing the average read depth of both whole exome and whole genome sequencing data) and B. SCHEMA Browser of SCZ associated rare variants. Note for each browser the conspicuous absence of any genetic variants (pathogenic or benign) from low read-depth (dark) regions from exome and whole genome sequencing data, across five of seven exons. **Figure S5.** Modified browser views of *CORO1A *from A. GnomAD Browser of human genetic variation (showing the average read depth of both whole exome and whole genome sequencing data); B. SCHEMA Browser of SCZ associated rare variants and C. Autism Sequencing Consortium Browser of rare variants. Note for each browser the conspicuous absence of any genetic variants (pathogenic or benign) from low read-depth (dark) regions from exome and whole genome sequencing data, in particular exon 10. **Figure S5.** Modified browser views of *SHANK2 *from A. GnomAD Browser of human genetic variation (showing the average read depth of both whole exome and whole genome sequencing data); B. SCHEMA Browser of SCZ associated rare variants and C. Autism Sequencing Consortium Browser of rare variants. Note for each browser the conspicuous absence of any genetic variants (pathogenic or benign) from low read-depth (dark) regions from exome and whole genome sequencing data. **Figure S6.** Modified browser views of *ARX *from A. GnomAD Browser of human genetic variation (showing the average read depth of both whole exome and whole genome sequencing data); B. SCHEMA Browser of SCZ associated rare variants and C. Autism Sequencing Consortium Browser of rare variants. Note for each browser the conspicuous absence of any genetic variants (pathogenic or benign) from low read-depth (dark) regions from exome and whole genome sequencing data, in particular exon 2. **Figure S7.** Modified browser views of *CASZ1 *from A. GnomAD Browser of human genetic variation (showing the average read depth of both whole exome and whole genome sequencing data); B. SCHEMA Browser of SCZ associated rare variants and C. Autism Sequencing Consortium Browser of rare variants. Note for each browser the conspicuous absence of any genetic variants (pathogenic or benign) from low read-depth (dark) regions from exome and whole genome sequencing data, in particular exons 9, 18 (alternatively spliced) and 19. **Figure S8.** Percentage of genes from SFARI (Score 1, Score 2, Score 3, Syndromic or absent from SFARI) with dark regions, stratified by ASC association q-values (less than 0.1; 0.1 to 0.3, greater than 0.3). This table shows that the SFARI genes with the greatest enrichment of dark gene bodies are those categorised as either Score 2 (High Confidence) or Syndromic, with ASC association q-values > 0.3.

## Data Availability

All data generated or analysed during this study are included in this published article (and its Additional files).
