# Supplementary material for: Investigating the dark-side of the genome: a barrier to human disease variant discovery?
Source: Biol Res. 2023 Jul 20;56:42. doi: 10.1186/s40659-023-00455-0 (PMC10357705; doi:10.1186/s40659-023-00455-0)
Supplement: Supplementary file 1 — Additional file 1: Methods S1. Table S1. GO enrichment analysis for the Schizophrenia dark genes vs genome generated 17 FDR significant GO terms. Table S2. GO enrichment analysis for the BMI dark genes vs rest of the genome generated 228 FDR significant GO terms, of which the top 30 are presented in this table. Table S3. GO enrichment analysis for the MDD GWAS genes with dark regions vs rest of the genome returned 45 FDR significant GO terms, of which the top 30 are represented in this table. Table S4. Results of the GO enrichment for the Cholesterol GWAS dark vs rest of genome, showing the top 30 enriched GO terms (out of 433 terms with p-value< 0.05, but not FDR significant). Table S5. Results of the GO enrichment for the Crohn’s dark vs genome, showing the top 30 enriched GO terms (out of 438 terms with p-value< 0.05, but not FDR significant). Table S6. Results of the GO enrichment for the ASD dark vs genome, showing the top 30 enriched GO terms (out of 106 terms with p-value< 0.05, but not FDR significant). Table S7. Results of the GO enrichment for the ALS GWAS dark genes vs rest of the genome, showing the top 30 enriched GO terms (out of 185 terms with p-value< 0.05 but not FDR significant). Table S8. Results of the GO enrichment for the BD GWAS dark genes vs rest of the genome, showing the top 30 enriched GO terms (out of 160 terms with p-value< 0.05 but not FDR significant). Table S9. SCZ darkCDS vs genome. Table S10. Results of the GO enrichment for the Schizophrenia dark CDS vs remaining GRL genes. No terms were FDR significant, however, five terms with p< 0.05 were returned, all related to brain development. Table S11. BMIdarkCDS vs genome Table S12. Top 20 GO terms for the BMI dark CDS vs remaining GRL genes. No FDR significant terms, however, 35 terms with p< 0.05 were returned. Table S13. MDDdarkCDS vs genome. Table S14. Top 20 GO terms for the MDD GWAS genes with dark CDS vs remaining GWAS genes. In total 40 terms (p< 0.05 but not FDR significant) wer [file 40659_2023_455_MOESM1_ESM.docx]

Additional Information

*Investigating the dark-side of the genome: a barrier to human disease variant discovery?* Ryan & Corvin

Table of Contents

Additional file [Methods 1](#_Toc113546619)

[Dark regions and dark genes: 1](#_Toc113546620)

[FUMA GWAS summary data: 1](#_Toc113546621)

[Dark regions and dark genes in GWAS: 1](#_Toc113546622)

[GWAS gene ontology enrichment: 2](#_Toc113546623)

[Additional Information: 2](#_Toc113546624)

[Project summary 2](#_Toc113546625)

[Gene ontology enrichment analysis of dark GRL: 3](#_Toc113546626)

[Whole exome sequencing datasets: 4](#_Toc113546627)

[**SCHEMA: 4**](#_Toc113546628)

[**ASC: 4**](#_Toc113546629)

[**SFARI: 5**](#_Toc113546630)

[Tables 6](#_Toc113546631)

[Figures 15](#_Toc113546632)

[References: 24](#_Toc113546633)

# Methods S1

## Dark regions and dark genes:

The list of dark regions in Hg37 was taken from the file *illuminaRL100.b37.dark-merged.bed* provided by Ebbert et al. (1) from their Github account (<https://github.com/mebbert/Dark_and_Camouflaged_genes>). This file contains the full set of 63,727 dark regions, covering 38.7Mb of the genome. The list of dark genes was taken from *IlluminaRL100.dark.gene.percentage.dark.txt* (Ebbert et al., 2019, Additional File 2). This file contains 6,054 genes, describing the percentage of each gene that is dark, stratified by gene body element (perc_CDS: percentage of protein coding sequence bases that are dark; perc_UTR: percentage of untranslated region bases that are dark; perc_exon: percentage of exonic bases that are dark; perc_intron: percentage of intronic bases that are dark; perc_total: percent of total gene that is dark). Of these, 749 genes have dark CDS (593 > 5% dark CDS; 424 > 20% dark CDS).

## FUMA GWAS summary data:

GWAS data was extracted from the FUMA database of public results, analysed on the FUMA platform, providing information on annotation, prioritization, visualization and interpretation of GWAS datasets (2-4). Eight GWAS were selected representing a range of diseases, disorders and complex traits as well as a range of numbers of gws loci. For comparability, all eight GWAS were selected based on their use of the following parameters: leadP (5e-8); gwasP (0.05); r2 (0.6); refpanel (1KG/Phase3); pop (EUR); refSNPs (1); mergeDist (250).

## Dark regions and dark genes in GWAS:

The overlap between dark regions and GWAS genome wide significant loci (Genomic Risk Loci (GRL)) was calculated using bedtools intersect using the following command format:

*bedtools intersect -a ASD_GWAS_FUMA_10_mapped_genes.txt -b illuminaRL100.b37.dark-merged.formatted.bed -wo > ASD_GWAS_FUMA_10_mapped_genes_dark_regions_overlap.bed*

For each GWAS study, the percentage of GRLs that are affected by dark regions was calculated as the total length of the dark regions within GRLs, which was then compared against the total length of the GWAS loci to calculate the percentage of GRL affected by dark regions.

Bedtools map was used to count all instances of dark genes overlapping mapped genes (MGs) within the GRLs. Where multiple dark regions overlapped the same gene, the IDs of the dark region were collapsed into a comma separated list:

*bedtools map -a ASD_GWAS_FUMA_10_mapped_genes_sort.txt -b illuminaRL100.b37.dark-merged.formatted_ID_sort.bed -c 4,4 -o count,collapse > ASD_GWAS_FUMA_10_mapped_genes_dark_regions_overlap_count_collapse.bed*

The amount of overlap between dark regions and MGs was also calculated using bedtools intersect:

*bedtools intersect -a ASD_GWAS_FUMA_10_mapped_genes.sort.txt -b illuminaRL100.b37.dark-merged.formatted_ID_sort.bed -wo > ASD_GWAS_FUMA_10_mapped_genes_dark_regions_overlap.bed*

The file *IlluminaRL100.dark.gene.percentage.dark.txt* contains the percentage overlap of dark regions with different gene body GENCODE biotypes (Frankish, Nucleic Acids Res. 2018) (coding regions (CDS); untranslated regions (UTR); exons; introns; and total percentage dark). This information was used to identify the subset of GWAS genes that had dark regions overlapping their coding regions (any dark, 5% dark, 20% dark).

## GWAS gene ontology enrichment:

From the Gene Ontology (GO) resource (<http://geneontology.org/>) (Ashburner et al., Nat Genet., 2000) the PANTHER Overrepresentation Test (Released 20210224; Mi et al., Nucleic Acids Res., 2019) was used to test for enrichment of biologically relevant GO terms within the subset of GWAS GRL genes annotated as overlapping dark regions compared against: i. the rest of the genome; and ii. the genes from GWAS GRLs without dark regions.

# Additional Information

## Project summary

We proposed to investigate whether dark regions could be affecting our ability to identify disease relevant variants, both when fine-mapping GRLs and when performing whole exome or whole genome rare variant association studies. Several new resources are now at our disposal to help achieve this goal. Firstly, the availability of summary data from GWAS studies through resources such as FUMA (2). GWAS studies identify regions of the genome, Genomic Risk Loci (GRLs), significantly associated with diseases/phenotypes, but without the ability to distinguish which genes/variants within these loci are actively contributing to disease-risk. By quantifying the amount of overlap between GRLs from recent GWAS and annotated dark regions, we can estimate the proportion of GRLs affected by dark regions. Furthermore, the GRL genes affected by dark regions can be tested for enrichment of disease relevant GO terms to assess potential biological impact of dark regions on our ability to identify putative risk genes when fine-mapping GWAS GRL. In addition to publicly available catalogues of GWAS studies, there are examples of rare-variant association studies, such as the Schizophrenia Exome Sequencing Meta-analysis (SCHEMA) consortium and the Autism Exome Sequencing consortium (ASC), which used whole exome sequencing (WES) data to identify genes with ultra-rare variants associated with disease. Genes identified from these rare variant association studies can similarly be tested for overlap with dark regions.

## Gene ontology enrichment analysis of dark GRL:

Gene ontology enrichment analysis using the PANTHER GO enrichment tool (5) was used to assess the potential functional impact of the dark genes from the GRLs. For each GWAS study the genes with dark regions from the GRLs were compared against the remaining genes from across the genome. Using this approach, the three largest GWAS studies (SCZ, BMI and MDD) all returned FDR-significant GO terms. SCZ had 17 FDR-significant GO terms, including terms related to neurogenesis; synapse; and calcium ion transport (Table S1). BMI had 228 FDR-significant terms, including terms related to regionalization; cell part morphogenesis; brain development; animal organ morphogenesis; regulation of protein localization; regulation of transport; positive regulation of transcription by RNA polymerase II; negative regulation of signal transduction; movement of cell or subcellular component; cellular catabolic process; organic substance catabolic process; establishment of localization in cell; phosphate-containing compound metabolic process, etc (Table S2). MDD had 45 FDR sig terms including those related to neurogenesis; synaptic function; neurotransmitter transport; and nervous system development (Table S3).

While cholesterol had no FDR-significant GO terms, the top terms included many terms related to cholesterol transport, and lipid and fatty acid biosynthesis and metabolism (Table S4). Similarly, the Crohn’s disease analysis returned relevant GO terms including immune response, cytokine activity, receptors and signalling, and glutamine metabolism (Table S5), all previously reported by Garza-Hernandez et al. (6). A number of the top enriched GO terms for ASD were related to glucose (regulation of detection of glucose; negative regulation of transcription by glucose, etc.) (Table S6), which have recently been implicated as a risk factor for ASD (7)manco (8). Other top terms were related to embryo development; cell-cell adhesion; neuron differentiation, transcription regulation, signalling pathways (9, 10). Top GO terms for ALS included membrane trafficking, signal transduction and ion channel transport; autophagy; central nervous system and oligo-dendrite development; dopaminergic neuron differentiation, neuron maturation, regulation of cell differentiation; central nervous system myelination and axon ensheathment and neuron axono-genesis (Table S7). Several of these terms were recently implicated in two independent GO enrichment analyses of ALS patients (11, 12). BD returned 160 terms with p-value< 0.05, with the top terms including terms related to piRNA biosynthetic process; CDP-diacylglycerol biosynthetic and metabolic process; regulation of postsynaptic neurotransmitter receptor activity; regulation of neurotransmitter receptor activity (Table S8)(13-15). Diacylglycerol kinase (DGK) isozymes have been reported to be involved in many physiological events, including cell proliferation and migration, glucose intake, immunity and neuronal network construction, and have been implicated in the pathogenesis of a wide variety of diseases including BD (16-20).

For the SCZ, BMI and MDD GWAS’s a comparison of the subset of GRL genes with dark CDS against i. the genome and ii. the remaining GRL genes (not-dark GRL genes)(Supplementary Tables 9-14) further refined the biological relevance of the GO terms identified (p-value < 0.05, but not FDR-significant). When comparing the dark CDS GRL genes vs the remaining GRL genes, SCZ had 5 enriched terms (striatal medium spiny neuron differentiation; striatum development; sub-pallium development; forebrain neuron differentiation; and forebrain generation of neurons); BMI had 32 terms, including terms previously associated with BMI (SMAD protein phosphorylation (21); sex determination; steroid biosynthesis; muscle morphogenesis; transmembrane receptor protein serine/threonine kinase signaling pathway (22); and GABAergic synaptic transmission (23); and MDD had 37 enriched terms (including presynaptic active zone assembly; protein localization to synapse; pre-synapse organization; pre-synapse assembly).

## Whole exome sequencing datasets:

To investigate the impact of dark regions on the discovery of rare variants from WES studies we looked at the overlap of dark regions with the coding parts of genes from two well-powered rare variant association studies: SCHEMA (schizophrenia exome sequencing project, (24) and ASC (autism exome sequencing project)(9). The genes with at least 5% dark CDS regions were assessed for other evidence of disease function in the literature.

### SCHEMA:

Of the 928 genes from SCHEMA with p<0.05, 222 were found to overlap dark regions with 22 of these having at least partially dark CDS regions (Table S15). Of the 10 dark genes found to have >5% CDS regions dark, six show evidence of from the literature of being expressed in the brain and having a neurodevelopmental or psychiatric function: *RAC3*; *TRAPPC10;* UBE2L3; *FAM86B1; SHANK3*; and *C4B*. *RAC3* is 7.3% dark (Figure S1) and has been shown to play a role in the development of GABAergic interneurons (25) and has been associated with neurodevelopmental disorders (26, 27). The *TRAPPC10* gene is 16% dark (Figure S2), is highly expressed in the cerebellum and has been suggested to be a candidate gene for intellectual disability , possibly through its role as a component of a transport protein particle complex (along with TRAPPC9 and TRAPPC2) that is involved in secretory and endocytic pathways (28). *UBE2L3* is 30% dark (Figure S3) and has been highlighted as a candidate gene for comorbid psychiatric and autoimmune disorders from a study that utilised pair-wise genetic correlations using the linkage disequilibrium score regression (LDSC) using GWAS summary statistics (29). Dysregulation of *UBE2L3* gene expression was also seen in blood of first episode psychosis patients compared against healthy controls (30). UBE2L3 was also shown to be down-regulated in a gene expression study of post-mortem brain tissue samples from SCZ patients (31). *FAM86B1* is 12.1% dark (Figure S4) and lies within a rare pathogenic CNV seen a patient with ID (32). This gene is in a GWAS locus implicated in Neuroticism (33). *SHANK3* and *C4B* have been described in detail in the main text.

### ASC:

Of the 102 ASD associated genes from the ASC (9), 46 overlap dark regions. Of these, four have dark CDS, two of which (*CORO1A* and *SHANK3*) are at least 5% dark CDS (Table S16). 36 of 116 genes with q-value >0.1 < 0.3 are partially dark (4 with dark CDS; none >5% dark CDS). 2982 of the 17127 genes with q-value > 0.3 are partially dark (322 with dark CDS; 214 >5% dark CDS). *CORO1A* is 7% dark (Figure S4), is also a SFARI Score 1 gene and was associated with SCZ in a recent GWAS (34). Of additional note, *SHANK2* which is 3.5% dark (Figure S5), is another ASD associated gene from the ASC study, is related to SHANK3 and has been implicated in a number of independent studies as being a ASD risk gene (35, 36). Like *SHANK3*, *SHANK2* encodes a synaptic scaffold protein and plays a role in synaptic plasticity and neuronal morphogenesis (37).

### SFARI:

SFARI Gene 3.0 integrates different kinds of genetic data with supplementary data and annotations to categorise genes based on the evidence supporting their link to autism (38). The SFARI scores are: Score 1 (High Confidence; 213 genes); Score 2 (Strong Candidate; 682 genes); Syndromic (ASD with co-morbid phenotypes; 153 genes) and Score 3 (Suggestive Evidence: 84 genes). Of the 213 genes with a SFARI Score 1, 93 were found to overlap dark regions, with ten of these genes having dark CDS regions (Table S17) and four being >5% dark CDS (*ARX***,** *CASZ1*, *CORO1A* and *SHANK3*). *ARX* is 12.8% dark (Figure S6) and is known to be expressed in GABAergic neuronal progenitors during human brain development (39, 40) and has been suggested to play a role in GABAergic dysfunction in schizophrenia (41). CASZ1 is 5.9% dark (Figure S7) and encodes a zinc finger transcription factor that has been suggested to play a role in neural fate-determination and neurogenesis (42). CASZ1 was recently found to be associated with ASD in a recent large-scale sequencing study (10) and has previously been associated with neurodevelopmental disorders (43).

Across the full set of SFARI genes we found an enrichment of dark regions in Score 2 and Syndromic (ASD with co-morbid phenotypes) genes with ASC q-values> 0.3, suggesting that some candidate genes for ASD may not perform well in genetic association studies due to their gene bodies being partially dark to sequencing (Figure S8).

# Tables

Full versions of Tables S1-15 can be found in the accompanying excel file.

Table S1. GO enrichment analysis for the Schizophrenia dark genes vs genome generated 17 FDR significant GO terms.

Table S2. GO enrichment analysis for the BMI dark genes vs rest of the genome generated 228 FDR significant GO terms, of which the top 30 are presented in this table.

Table S3. GO enrichment analysis for the MDD GWAS genes with dark regions vs rest of the genome returned 45 FDR significant GO terms, of which the top 30 are represented in this table.

 Table S4. Results of the GO enrichment for the Cholesterol GWAS dark vs rest of genome, showing the top 30 enriched GO terms (out of 433 terms with p-value< 0.05, but not FDR significant).

Table S5. Results of the GO enrichment for the Crohn’s dark vs genome, showing the top 30 enriched GO terms (out of 438 terms with p-value< 0.05, but not FDR significant).

Table S6. Results of the GO enrichment for the ASD dark vs genome, showing the top 30 enriched GO terms (out of 106 terms with p-value< 0.05, but not FDR significant).

Table S7. Results of the GO enrichment for the ALS GWAS dark genes vs rest of the genome, showing the top 30 enriched GO terms (out of 185 terms with p-value< 0.05 but not FDR significant).

Table S8. Results of the GO enrichment for the BD GWAS dark genes vs rest of the genome, showing the top 30 enriched GO terms (out of 160 terms with p-value< 0.05 but not FDR significant).

Table S10. Results of the GO enrichment for the Schizophrenia dark CDS vs remaining GRL genes. No terms were FDR significant, however, five terms with p< 0.05 were returned, all related to brain development.

Table S12. Top 20 GO terms for the BMI dark CDS vs remaining GRL genes. No FDR significant terms, however, 35 terms with p< 0.05 were returned.

Table S14. Top 20 GO terms for the MDD GWAS genes with dark CDS vs remaining GWAS genes. In total 40 terms (p< 0.05 but not FDR significant) were returned.

 Table S15. All 22 SCHEMA genes with p<0.05 with at least partially dark CDS regions of which ten genes have >5% dark CDS.

Table S16. Subset of ASD associated genes from the ASC which have dark CDS regions, of which two genes have >5% dark CDS.

Table S17. SFARI Score 1 (High Confidence) genes with dark CDS regions, of which four genes have >5% dark CDS.

# Figures

Figure S1. Modified browser views of *RAC3* from A. GnomAD Browser of human genetic variation (showing the average read depth of both whole exome and whole genome sequencing data) and B. SCHEMA Browser of SCZ associated rare variants. Note for each browser the conspicuous absence of any genetic variants (pathogenic or benign) from low read-depth (dark) regions from exome and whole genome sequencing data, in particular for exon 1.

Figure S2. Modified browser views of *TRAPPC10* from A. GnomAD Browser of human genetic variation (showing the average read depth of both whole exome and whole genome sequencing data) and B. SCHEMA Browser of SCZ associated rare variants.

Figure S3. Modified browser views of *UBE2L3* from A. GnomAD Browser of human genetic variation (showing the average read depth of both whole exome and whole genome sequencing data) and B. SCHEMA Browser of SCZ associated rare variants. Note for each browser the conspicuous absence of any genetic variants (pathogenic or benign) from low read-depth (dark) regions from exome and whole genome sequencing data, in particular for exon 1.

Figure S4. Modified browser views of *FAM86B1* from A. GnomAD Browser of human genetic variation (showing the average read depth of both whole exome and whole genome sequencing data) and B. SCHEMA Browser of SCZ associated rare variants. Note for each browser the conspicuous absence of any genetic variants (pathogenic or benign) from low read-depth (dark) regions from exome and whole genome sequencing data, across five of seven exons.

Figure S5. Modified browser views of *CORO1A* from A. GnomAD Browser of human genetic variation (showing the average read depth of both whole exome and whole genome sequencing data); B. SCHEMA Browser of SCZ associated rare variants and C. Autism Sequencing Consortium Browser of rare variants. Note for each browser the conspicuous absence of any genetic variants (pathogenic or benign) from low read-depth (dark) regions from exome and whole genome sequencing data, in particular exon 10.

Figure S5. Modified browser views of *SHANK2* from A. GnomAD Browser of human genetic variation (showing the average read depth of both whole exome and whole genome sequencing data); B. SCHEMA Browser of SCZ associated rare variants and C. Autism Sequencing Consortium Browser of rare variants. Note for each browser the conspicuous absence of any genetic variants (pathogenic or benign) from low read-depth (dark) regions from exome and whole genome sequencing data.

Figure S6. Modified browser views of *ARX* from A. GnomAD Browser of human genetic variation (showing the average read depth of both whole exome and whole genome sequencing data); B. SCHEMA Browser of SCZ associated rare variants and C. Autism Sequencing Consortium Browser of rare variants. Note for each browser the conspicuous absence of any genetic variants (pathogenic or benign) from low read-depth (dark) regions from exome and whole genome sequencing data, in particular exon 2.

Figure S7. Modified browser views of *CASZ1* from A. GnomAD Browser of human genetic variation (showing the average read depth of both whole exome and whole genome sequencing data); B. SCHEMA Browser of SCZ associated rare variants and C. Autism Sequencing Consortium Browser of rare variants. Note for each browser the conspicuous absence of any genetic variants (pathogenic or benign) from low read-depth (dark) regions from exome and whole genome sequencing data, in particular exons 9, 18 (alternatively spliced) and 19.

**Figure S8.** Percentage of genes from SFARI (Score 1, Score 2, Score 3, Syndromic or absent from SFARI) with dark regions, stratified by ASC association q-values (less than 0.1; 0.1 to 0.3, greater than 0.3). This table shows that the SFARI genes with the greatest enrichment of dark gene bodies are those categorised as either Score 2 (High Confidence) or Syndromic, with ASC association q-values > 0.3.

# References

1. Ebbert MTW, Jensen TD, Jansen-West K, Sens JP, Reddy JS, Ridge PG, et al. Systematic analysis of dark and camouflaged genes reveals disease-relevant genes hiding in plain sight. Genome Biol. 2019;20(1):97.

2. Watanabe K, Taskesen E, van Bochoven A, Posthuma D. Functional mapping and annotation of genetic associations with FUMA. Nat Commun. 2017;8(1):1826.

3. Ashburner M, Ball CA, Blake JA, Botstein D, Butler H, Cherry JM, et al. Gene ontology: tool for the unification of biology. The Gene Ontology Consortium. Nat Genet. 2000;25(1):25-9.

4. Gene Ontology C. The Gene Ontology resource: enriching a GOld mine. Nucleic Acids Res. 2021;49(D1):D325-D34.

5. Mi H, Muruganujan A, Ebert D, Huang X, Thomas PD. PANTHER version 14: more genomes, a new PANTHER GO-slim and improvements in enrichment analysis tools. Nucleic Acids Res. 2019;47(D1):D419-D26.

6. Garza-Hernandez D, Sepulveda-Villegas M, Garcia-Pelaez J, Aguirre-Gamboa R, Lakatos PL, Estrada K, et al. A systematic review and functional bioinformatics analysis of genes associated with Crohn's disease identify more than 120 related genes. BMC Genomics. 2022;23(1):302.

7. Hoirisch-Clapauch S, Nardi AE. Autism spectrum disorders: let's talk about glucose? Transl Psychiatry. 2019;9(1):51.

8. Manco M, Guerrera S, Rava L, Ciofi Degli Atti M, Di Vara S, Valeri G, et al. Cross-sectional investigation of insulin resistance in youths with autism spectrum disorder. Any role for reduced brain glucose metabolism? Transl Psychiatry. 2021;11(1):229.

9. Satterstrom FK, Kosmicki JA, Wang J, Breen MS, De Rubeis S, An JY, et al. Large-Scale Exome Sequencing Study Implicates Both Developmental and Functional Changes in the Neurobiology of Autism. Cell. 2020;180(3):568-84 e23.

10. Trost B, Thiruvahindrapuram B, Chan AJS, Engchuan W, Higginbotham EJ, Howe JL, et al. Genomic architecture of Autism Spectrum Disorder from comprehensive whole-genome sequence annotation. medRxiv. 2022:2022.05.05.22274031.

11. Kotni MK, Zhao M, Wei DQ. Gene expression profiles and protein-protein interaction networks in amyotrophic lateral sclerosis patients with C9orf72 mutation. Orphanet J Rare Dis. 2016;11(1):148.

12. Saez-Atienzar S, Bandres-Ciga S, Langston RG, Kim JJ, Choi SW, Reynolds RH, et al. Genetic analysis of amyotrophic lateral sclerosis identifies contributing pathways and cell types. Sci Adv. 2021;7(3).

13. Ashok AH, Marques TR, Jauhar S, Nour MM, Goodwin GM, Young AH, et al. The dopamine hypothesis of bipolar affective disorder: the state of the art and implications for treatment. Mol Psychiatry. 2017;22(5):666-79.

14. Yuksel C, Ongur D. Magnetic resonance spectroscopy studies of glutamate-related abnormalities in mood disorders. Biol Psychiatry. 2010;68(9):785-94.

15. Blacker CJ, Lewis CP, Frye MA, Veldic M. Metabotropic glutamate receptors as emerging research targets in bipolar disorder. Psychiatry Res. 2017;257:327-37.

16. Baum AE, Akula N, Cabanero M, Cardona I, Corona W, Klemens B, et al. A genome-wide association study implicates diacylglycerol kinase eta (DGKH) and several other genes in the etiology of bipolar disorder. Mol Psychiatry. 2008;13(2):197-207.

17. Squassina A, Manchia M, Congiu D, Severino G, Chillotti C, Ardau R, et al. The diacylglycerol kinase eta gene and bipolar disorder: a replication study in a Sardinian sample. Mol Psychiatry. 2009;14(4):350-1.

18. Kittel-Schneider S, Lorenz C, Auer J, Weissflog L, Reif A. DGKH genetic risk variant influences gene expression in bipolar affective disorder. J Affect Disord. 2016;198:148-57.

19. Sakane F, Hoshino F, Murakami C. New Era of Diacylglycerol Kinase, Phosphatidic Acid and Phosphatidic Acid-Binding Protein. Int J Mol Sci. 2020;21(18).

20. Saxena A, Scaini G, Bavaresco DV, Leite C, Valvassori SS, Carvalho AF, et al. Role of Protein Kinase C in Bipolar Disorder: A Review of the Current Literature. Mol Neuropsychiatry. 2017;3(2):108-24.

21. Seong HA, Manoharan R, Ha H. Smad proteins differentially regulate obesity-induced glucose and lipid abnormalities and inflammation via class-specific control of AMPK-related kinase MPK38/MELK activity. Cell Death Dis. 2018;9(5):471.

22. Mugabo Y, Lim GE. Scaffold Proteins: From Coordinating Signaling Pathways to Metabolic Regulation. Endocrinology. 2018;159(11):3615-30.

23. Xia G, Han Y, Meng F, He Y, Srisai D, Farias M, et al. Reciprocal control of obesity and anxiety-depressive disorder via a GABA and serotonin neural circuit. Mol Psychiatry. 2021;26(7):2837-53.

24. Singh T, Poterba T, Curtis D, Akil H, Al Eissa M, Barchas JD, et al. Rare coding variants in ten genes confer substantial risk for schizophrenia. Nature. 2022;604(7906):509-16.

25. de Curtis I. Roles of Rac1 and Rac3 GTPases during the development of cortical and hippocampal GABAergic interneurons. Front Cell Neurosci. 2014;8:307.

26. Costain G, Callewaert B, Gabriel H, Tan TY, Walker S, Christodoulou J, et al. De novo missense variants in RAC3 cause a novel neurodevelopmental syndrome. Genet Med. 2019;21(4):1021-6.

27. Hiraide T, Kaba Yasui H, Kato M, Nakashima M, Saitsu H. A de novo variant in RAC3 causes severe global developmental delay and a middle interhemispheric variant of holoprosencephaly. J Hum Genet. 2019;64(11):1127-32.

28. Santos-Cortez RLP, Khan V, Khan FS, Mughal ZU, Chakchouk I, Lee K, et al. Novel candidate genes and variants underlying autosomal recessive neurodevelopmental disorders with intellectual disability. Hum Genet. 2018;137(9):735-52.

29. Tylee DS, Sun J, Hess JL, Tahir MA, Sharma E, Malik R, et al. Genetic correlations among psychiatric and immune-related phenotypes based on genome-wide association data. Am J Med Genet B Neuropsychiatr Genet. 2018;177(7):641-57.

30. Leirer DJ, Iyegbe CO, Di Forti M, Patel H, Carra E, Fraietta S, et al. Differential gene expression analysis in blood of first episode psychosis patients. Schizophr Res. 2019;209:88-97.

31. Huang KC, Yang KC, Lin H, Tsao TT, Lee SA. Transcriptome alterations of mitochondrial and coagulation function in schizophrenia by cortical sequencing analysis. BMC Genomics. 2014;15 Suppl 9:S6.

32. Fry AE, Rees E, Thompson R, Mantripragada K, Blake P, Jones G, et al. Pathogenic copy number variants and SCN1A mutations in patients with intellectual disability and childhood-onset epilepsy. BMC Med Genet. 2016;17(1):34.

33. Hill WD, Weiss A, Liewald DC, Davies G, Porteous DJ, Hayward C, et al. Genetic contributions to two special factors of neuroticism are associated with affluence, higher intelligence, better health, and longer life. Mol Psychiatry. 2020;25(11):3034-52.

34. Trubetskoy V, Pardinas AF, Qi T, Panagiotaropoulou G, Awasthi S, Bigdeli TB, et al. Mapping genomic loci implicates genes and synaptic biology in schizophrenia. Nature. 2022;604(7906):502-8.

35. Zaslavsky K, Zhang WB, McCready FP, Rodrigues DC, Deneault E, Loo C, et al. SHANK2 mutations associated with autism spectrum disorder cause hyperconnectivity of human neurons. Nat Neurosci. 2019;22(4):556-64.

36. Lee YS, Yu NK, Chun J, Yang JE, Lim CS, Kim H, et al. Identification of a novel Shank2 transcriptional variant in Shank2 knockout mouse model of autism spectrum disorder. Mol Brain. 2020;13(1):54.

37. Jiang YH, Ehlers MD. Modeling autism by SHANK gene mutations in mice. Neuron. 2013;78(1):8-27.

38. Abrahams BS, Arking DE, Campbell DB, Mefford HC, Morrow EM, Weiss LA, et al. SFARI Gene 2.0: a community-driven knowledgebase for the autism spectrum disorders (ASDs). Mol Autism. 2013;4(1):36.

39. Friocourt G, Parnavelas JG. Mutations in ARX Result in Several Defects Involving GABAergic Neurons. Front Cell Neurosci. 2010;4:4.

40. Lim Y, Cho IT, Shi X, Grinspan JB, Cho G, Golden JA. Arx Expression Suppresses Ventralization of the Developing Dorsal Forebrain. Sci Rep. 2019;9(1):226.

41. Nakamura JP, Schroeder A, Hudson M, Jones N, Gillespie B, Du X, et al. The maternal immune activation model uncovers a role for the Arx gene in GABAergic dysfunction in schizophrenia. Brain Behav Immun. 2019;81:161-71.

42. Liu Z, Naranjo A, Thiele CJ. CASZ1b, the short isoform of CASZ1 gene, coexpresses with CASZ1a during neurogenesis and suppresses neuroblastoma cell growth. PLoS One. 2011;6(4):e18557.

43. Wang T, Hoekzema K, Vecchio D, Wu H, Sulovari A, Coe BP, et al. Large-scale targeted sequencing identifies risk genes for neurodevelopmental disorders. Nat Commun. 2020;11(1):4932.
